# Note on the Use of Chloride of Methyl

**Published:** 1888-08

**Authors:** Wm. M. Thallon


### NOTE ON THE USE OF CHLORIDE OF METHYL.

BY WM. M. THALLON, M. D.

Chloride of methyl was introduced to the profession in 1848 by M. Debove, of Paris. He reported a number of cases before the Medical Society of the Hospitals of Paris, in which he had used this agent externally in the treatment of neuralgias, both primary and secondary. His results were so remarkable that several of the French observers at once had recourse to his methods, and a series of clinical reports have since appeared in the French medical journals. The uniformity of results obtained is quite striking, most of the cases, even inveterate sciaticas of years' standing, being cured, while all are reported as being more or less relieved. My own attention was directed to this remedy by the manufacturers, Messrs. Brigounet & Noville, of Paris, who sent me two syphons filled with chloride of methyl, with the request to try it and report on the results. I sent one of the syphons to Dr. E. C. Seguin, of New York, whose experience so far, I believe, has been favorable, but which he will doubtless himself report fully hereafter.

The first case I tried chloride of methyl in, was a patient who had been a martyr to neuralgia in various forms for years, and who, in consequence, had had recourse to morphine. I found her suffering from intense pain, located apparently in the musculo-spiral nerve of one arm. There were numerous painful points on pressure, especially where the nerve enters the muscular septum. I tried various remedies—aconitia, arsenic, quinine, phosphorus, cannabis indica, atropine, all in efficient doses, without benefit. Then I used the constant current and sinapisms also, without affording relief. I was afraid I would be driven back to the use of morphine. At this juncture I received the syphon of chloride of methyl. I at once tried it, and the very first application gave great relief. The patient said she slept better that night than for weeks. I subsequently made slighter applications. The patient was so much better that she went to the country; this was in July. Last week I saw her again, and she stated that though she had had other pain in the shoulder, she had had no recurrence in the forearm.

The next case was one in which severe and persistent pain followed a mild attack of milk-leg. While the area of pain could not be nearly as accurately localized as in the preceding case, it was mainly referred to the cutaneous branches of the anterior crura nerve. The applications made were very extensive in area, but not very intense in degree. Unfortunately, my supply of methyl gave out on the second day, and a complete cure was not effected, still the relief obtained was very marked. Other means, such, as aconitia internally, the local use of the oleates of morphine and atropine, the constant current, lead and opium wash, sinapisms and extensive warmth, produced by wrapping the limb in heated cotton batting, had all been used previously, without affording any relief. The first change for the better immediately followed the use of the chloride of methyl. Dr. C. Jewett, who twice saw this case in consultation with me, will endorse the peculiarly inveterate nature of the attack.

The third case was one of facial neuralgia, the patient having had repeated attacks, sometimes accompanied by an herpetic eruption in the first devision of the trigeminal nerve. Here a single application banished the pain, which has not since returned.

I should not have ventured to bring this matter before you with such meagre clinical data of my own, if it were not that the evidence of the French observers was more ample. Thus Debove, in his first paper, says: "My first patient treated was sent me by Dr. Dumonteil, and had a sciatica, which at different times had been fruitlessly treated by the actual cautery, and whom rest for one month during my hospital service had not in the least relieved. I applied the spray of chloride of methyl over the entire painful surface, from the hip to the external mallelous. One minute later, this patient, who had been unable to take a step, walked without limping, declaring himself cured. He was quite stupefied at his recovery, and I may confess my astonishment was not inferior to his. But I knew too much of marvellous therapeutic results due to coincidences and to the effects of imagination, and which the doctor does not again obtain on further trials, to be deceived. That is especially true of sciatica, which has actually been relieved by cauterizing the lobe of the ear. But, thanks to the frequency of this disease, I have been enabled to repeat my experiments with chloride of methyl, and I can affirm to-day that we are dealing here with a therapeutic agent, the effects of which are constant and instantaneous."

In his further remarks, Dr. Debove cites over a hundred cases in which he had successfully used this method. The experience of M. Tenneson, whom I know personally to be a careful and conscientious observer, though not so extended or striking, is noteworthy. He reports thirty-seven cases in all. Of these, ten were sciaticas, in seven of which there was immediate and complete cure of the pain; in three marked relief but only partial cure. In eleven cases of muscular rheumatism, nine were relieved of pain by one application of the spray, and two by two applications. In five cases of articular rheumatism, either acute or subacute, the relief of the pain was immediate, though the inflammatory swelling persisted and motion remained limited. Two cases of chronic articular rheumatism were markedly relieved of pain, and motion of the affected joints became more free. One case of periosteal pain of the sacrum in a tuberculous subject was relieved, and did not return during the few remaining weeks of his life.

Lastly, in seven cases of localized painful points—three in phthisis. one in purulent pleurisy, and three in acute lobar pneumonia—the spray brought complete relief.

I might quote sti.l more extensively from the published reports, but these cases are sufficient to show that we have here a therapeutic agent that deserves i..vestigation. I will now say a few words about the method of application.

The chloride of methyl, at ordinary temperature, is a gas which can be liquefied by extreme cold or pressure, or by combined cold and pressure. As used in medicine, it comes in the liquid state, in strong copper cylinders, under high pressure. If the pressure is removed, it expands and becomes a gas, the passage from the liquid to the gaseous state following the general physical law of absorption of sensible heat from the surrounding objects or air. This may be so intense as to produce a degree of cold in the object from which the heat is absorbed of 67° F. below zero.

This property of chloride of methyl has been utilized by the French microscopists in freezing specimens for cutting.

In this apparatus, in the top, is a vertical pin, moved by a screw. On unscrewing it slightly, the chloride of methl expands into the small chamber thus created; from this a lateral opening leads to the spray-tube, which is attached by a joint, allowing motion through a complete circle, by means of which the desired direction can be given to the spray. The calibre of the spray-tube is extremely fine, and by means of this screw, which opens or closes it, its coarseness and the degree of force with which it strikes the skin can be accurately regulated. There has been hitherto no source of supply in this country where chloride of methyl could be obtained, but Messrs. McKesson & Robbins inform me that they are negotiating with the proprietors of this article in Paris for its introduction into the United States.

The distance at which the end of the spray tube should be held from the skin is from eight to twelve inches. The length of time the application to any one area of skin should last, varies with its thickness, but should never exceed one second. It is better to spray a part twice moderately, than too intensely once. The danger of too prolonged an application is that you may get a blister, an eschar, or even a slough, just as in frost-bite. This should never occur with anything like reasonable care. The color of the skin offers a perfect guide, and it should be closely watched. The moment the skin becomes pale and hard, our object is attained, and the spray should cease. This palor of the skin is very rapidly followed by a more or less intense redness. This may persist from a few hours to several days, and some discoloration often lasts for weeks, but not permanently. Lastly, the most important practical point is, that as large a portion as possible of the cutaneous distribution of the affected nerve must be acted on.

These applications are to a certain degree painful; but owing to the rapidity and intensity of action of the cold, the initial pain is merely momentary. When the redness of the skin supervenes, there is more or less sensation of burning, but not as severe as that following the application of the actual cautery.

In conclusion, I would beg to draw your attention to the fact that this method of treatment is in no wise offered as a substitute for proper constitutional medication; it is merely a valuable adjunct. It seems to me especially valuable in that the methyl spray is a reliable substitute for that fearfully abused class of medicines —anodynes.

The principle involved in the use of intense cold as a counter-irritant in medicine is very old, but this method of applying it is new. I think, moreover, it possesses certain decided advantages over any other therapeutic agent of its kind that I am acquainted with. In the first place, with very little skill in manipulation its extent can be accurately regulated, so can its intensity. The rapidity of its action renders it far less painful as well as more efficient than the ether spray. I have tried both on myself, and can testify from painful personal experience. Its advantage over some other counter-irritants, such as cantharides, is that it can be so much more extensively applied and is not followed by a painful vesication or eschar, taking perhaps a week to heal. I think my neurological friends will bear me out, that counter-irritation for the relief of pain should not transcend a certain degree of intensity, and should be applied, as far as practicable, to the *entire* cutaneous distribution of the affected nerve, in order to obtain the greatest good. I am quite sure it is not enough to counter-irritate over the painful points of Valleix. I have convinced myself clinically, in using the actual cautery, that you have the best success by starting at the most distant peripheral point and working toward the centre. The same holds good for the use of the galvanic current in the treatment of neuralgia. It is this extensive adaptability that gives so much value to the spray of chloride of methyl.

I have confined myself in this brief note to the use of methyl chloride for the relief of pain, but if its counter-irritant properties are as reliable as I think them, I believe that its applicability is much wider. It would be hard to mention any therapeutic agency which has more thoroughly stood the test of long experience than counter-irritation. From time immemorial the testimony as to reliability and efficiency has been overwhelming. Many pathological reflex actions, such, for instance, as coughs, are markedly relieved by properly applied counter-irritants. I do not doubt that some such methods as this of spray of chloride of methyl will offer the doctor a better means of applying counter irritation than any other which we have at present at our command.—*Brooklyn Medical Journal.*

